# Tidier. e-sport; a recovery oriented intervention in forensic psychiatry

**DOI:** 10.1192/j.eurpsy.2021.1016

**Published:** 2021-08-13

**Authors:** L. Sørensen, H. Kennedy, B. Jensen, M. Terkildsen, R. Poulsen, M. Josefsen, A. Di Lieto

**Affiliations:** 1 Department Of Clinical Medicine, Aarhus University- Health, Aarhus N, Denmark; 2 Department Of Forensic Psychiatry, Aarhus University Hopsital Psychiatry, Aarhus N, Denmark; 3 Department Of Psychiatry, Trinity College- Dublin University, Dublin, Ireland; 4 National Forensic Mental Health Service, Central Mental Hospital Dundrum, Dundrum, Ireland

## Abstract

**Introduction:**

Recently video gaming, have attracted considerable attention for its possible beneficial therapeutic effects, the possibility for testing behavior in safe artificial environments and as a tool for professionals and patients to build specific competencies for the everyday life. Also, a substantial amount of research suggests that videogaming might improve the participants social and cognitive skills and emotional regulation. There is little or no evidence that videogaming increases long term aggression or leads to physical aggression. At a medium secure forensic psychiatric in-patient ward, the patients and staff engage in weekly E – Sport sessions (primarily counterstrike) to further the recovery process.

**Objectives:**

To provide a standardized description of how E-sport is organized and used in the recovery process among forensic psychiatric patients.

**Methods:**

The Template for Intervention Description and Replication (TIDieR) checklist and guide is widely used to in health research to describe interventions in clinical trials and other health research contexts. By use of TIDieR we describe a newly developed E-sport intervention, in which staff members and patients in a medium secure forensic psychiatric ward engage in weekly E-Sport sessions (primarily counterstrike) to improve patient–staff relationship.

**Results:**

The E-sport intervention is detailed by use of the 12 TIDieR items and practical experiences and insights will be described.

**Conclusions:**

This standardized and detailed description of how is used in a recovery-oriented process in forensic psychiatry can be used for future studies that wishes to implement the intervention or for research studies replicating the treatment.

**Conflict of interest:**

No significant relationships.

